# Clean Style Recovery and Utilization of Residual Nutrients in Effluents From Biohydrogen Production: *In Situ* Immobilization Based on Sodium Alginate

**DOI:** 10.3389/fbioe.2022.906968

**Published:** 2022-05-16

**Authors:** Fuke Ai, Yang Zhang, Xiaoni Fan, Yameng Li, Haorui Zhang, Yinggang Jiao, Quanguo Zhang, Cheng Yong, Jinfei Zhao, Francesco Petracchini, Valerio Paolini, Zhiping Zhang

**Affiliations:** ^1^ Key Laboratory of New Materials and Facilities for Rural Renewable Energy, MOA of China, Henan Agricultural University, Zhengzhou, China; ^2^ Institute of Agricultural Resources and Environment, Jiangsu Academy of Agricultural Sciences, Nanjing, China; ^3^ Key Laboratory of Modern Agricultural Engineering of Xinjiang Higher Education Institutions, Alar, China; ^4^ National Research Council of Italy- Institute of Atmospheric Pollution Research, Rome, Italy

**Keywords:** sodium alginate, slurry capsule, fermentative biohydrogen production, immobilization effect, fertilizer

## Abstract

Clean- and high-value recovery and reuse of the residue of biohydrogen production (biohydrogen slurry) is an urgent problem to be solved. In this study, sodium alginate (SA) gel was used to concentrate nutrients quickly *in situ* from biohydrogen slurry, which was prepared into gel microspheres (GMs), just like “capsule.” The immobilization and release efficiency of conventional and reverse spherification were investigated. Better immobilization and release efficiency were detected under the conventional spherification method. The effect of GM sizes and concentrations of SA and calcium chloride (CaCl_2_) was further studied in terms of sphericity factor, nutrient release, yield, encapsulation efficiency, and loading capacity. The best immobilization effect was obtained with a 1.6-mm syringe needle, 3.0 wt% SA, and 6 wt% CaCl_2_, in which the sphericity factor, nitrogen release, yield, nitrogen encapsulation efficiency, and nitrogen loading capacity reached to 0.047, 96.20, 77.68, 38.37, and 0.0476%, respectively. This process not only avoids environmental pollution from biohydrogen slurry but also uses them at a high value as a fertilizer to nourish the soil. The feasibility of “slurry capsule” preparation will realize the clean recovery and reuse of biohydrogen slurry, which provides a new idea for ecological protection and carbon neutral goals and has important significance for sustainable development.

## 1 Introduction

The resource utilization of biomass is an important measure to promote the green and sustainable development and reduce carbon emissions (Hameed et al., 2021). Agricultural waste recycling combined with green hydrogen production has become an important scientific innovation exploration (Lepage et al., 2021). Among several green hydrogen production pathways, fermentative biohydrogen production (FHP) is a promising one, which has a wide range of raw materials, high substrate utilization efficiency, and low energy consumption (Anwar et al., 2019). A large amount of fermentation wastewater (biohydrogen slurry) will be produced after the process of hydrogen production by fermentation. The biohydrogen slurry after hydrogen production is rich in not only nitrogen, phosphorus, potassium, and other mineral nutrients needed for plant growth but also some beneficial amino acids (Akhlaghi and Najafpour-Darzi, 2020). Some of the nutrients are obtained from the addition in the process of hydrogen production but not fully utilized, and the other part is obtained from the degradation of raw materials (Wang Y. et al., 2021). The process and composition of biohydrogen slurry and biogas slurry are similar. It has been reported that biogas slurry could be used as a liquid fertilizer to irrigate land, which can significantly promote nitrogen absorption by plants, increase crop yield, and improve soil structure (Zeng et al., 2022). But there are no reports about the utilization of biohydrogen slurry; instead, many researchers focused on the optimization and enhancement of the hydrogen production process (Zhu et al., 2021).

Compared with biogas slurry, biohydrogen slurry has higher VFAs and lower pH. From the angle of economy, discharging the tail liquid directly into the field seems to be most beneficial, but the absorption capacity of soil is limited and excessive biohydrogen slurry returning to the field may cause soil acidification and groundwater pollution, posing direct and indirect risks to human health (Li et al., 2021). The concentration of biohydrogen slurry is low, the water content is high, and excessive water content limits the direct return of biohydrogen slurry to the field. In addition, providing a large amount of nutrient elements for the plant at one time cannot achieve the maximum effect of nutrient elements because the demand for nutrient elements is different in different growth stages of the plant (Zhang et al., 2022). Therefore, it is necessary to explore a technology that can concentrate nutrients from the biohydrogen slurry and release nutrients slowly. By concentrating nutrients from the biohydrogen slurry, the storage and transportation of biohydrogen slurry can be reduced. *In situ* immobilization is the focus of recent research to immobilize the nutrients on the carrier in a special form by ion exchange, electrostatic attraction, physical adsorption, and carbonate precipitation, which is mainly used for the remediation of extensive contamination with heavy metals (Wang G. et al., 2021; Xie et al., 2022) and rapid *in situ* removal of residual antibiotics in aquaculture systems (Sha et al., 2022). Biochar, metal oxides, phosphate compounds, and silicon fertilizer are common immobilized carriers and show better fixation and sealing effect (Huang et al., 2020), preventing the fixed target precipitation from fixed carriers. But for the nutrients in biohydrogen slurry, the immobilized carrier should meet the requirement, which not only can concentrate nutrients from the biohydrogen slurry but also release nutrients slowly.

Recently, sodium alginate (SA) gel, as an environmentally friendly material, has been used in the field of environmental remediation because of its hydrophilicity and stability (Thakur et al., 2018). SA was used to fix magnesium-loaded bentonite to produce beads, which could effectively adsorb phosphate in water, and the beads could be used as slow-release fertilizer for the growth of mint (Xi et al., 2021). At present, there is no unified evaluation standard for slow-release fertilizer, and the method of comparison with the chemical fertilizer is generally used to evaluate its slow-release performance. The composite hydrogel modified by polyacrylamide with sodium alginate had higher pesticide-loading efficiency and sustained release performance and had a positive effect on the clean utilization of pesticides (Wang et al., 2019). Specifically, the environmental remediation mechanism of SA is mainly adsorption and slow release, that is, using the characteristics of SA to provide a stable condition for the inclusion so as to realize the ecological environment management *via* the character of the inclusion (Fernando et al., 2020; Gao et al., 2020).

Hence, considering the physicochemical characters of biohydrogen slurry and nutrient release requirement, the SA immobilization method, which is not only a conventional fix method but also a novel utilization pathway for fixing the beneficial substances in the biohydrogen slurry produced from the FHP process, was discussed. Using SA as the carrier, gel microspheres (GMs) are rapidly formed by combining with polyvalent cations under extremely mild conditions (Tokarev and Minko, 2010). The production of GMs can realize the clean utilization of biohydrogen slurry. For its fixation of biohydrogen slurry, active substances, such as nutrients, proteins, and amino acids, can be embedded by SA, and the composition retention and effectiveness can be maximized. Moreover, the GMs are easier to store and transport and have the effect of slow release of nutrients, just like the “slurry capsule” that can be used for soil remediation, nutrient control, and treatment of other soil problems (Bennacef et al., 2021). This process not only avoids environmental pollution from biohydrogen slurry but also uses them at a high value as a fertilizer to nourish the soil.

## 2 Material and Methods

### 2.1 Chemicals and Materials

Five types of biohydrogen slurry were collected from the following experiments, dark-fermentative biohydrogen production from alfalfa (ADF), dark-fermentative biohydrogen production from corn straw (SCDF), photo-fermentative biohydrogen production from corncob (CLF), photo-fermentative biohydrogen production from co-digestion of cow dung and corn straw (CCLF), and photo-fermentative biohydrogen production from corn straw with the biochar additive (CBLF). The procedures of the FHP process are mentioned in the previous literature (Garcia-Depraect et al., 2021; Zhang et al., 2021). The composition of the five biohydrogen slurries and mock biohydrogen slurry (MBS) is shown in Table 1.

**TABLE 1:** Composition of the five biohydrogen slurries.

Biohydrogen slurry	Nutritive element (mg/L)					Amino acid (mg/L)		
	N	P	K	Mg	Ca	Phe	Pro	Arg
ADF	2057.840	70.339	2636.114	74.258	255.463	6.661	2.370	2.772
CCLF	71.543	68.939	154.689	49.008	99.138	0.329	0.141	0.037
CBLF	148.683	39.889	1028.864	22.525	55.288	0.428	0.173	0.269
SCDF	628.430	286.114	740.614	83.733	157.388	3.388	0.996	2.913
CLF	104.893	27.189	1274.864	27.658	87.488	0.496	0.489	0.485
MBS	2000.000	100.000	2200.000	——	——	7.000	3.000	3.000

SA is obtained from Fuchen (Tianjin) Chemical Reagent Co., LTD. Anhydrous calcium chloride (CaCl_2_) is obtained from Kemiou (Tianjin) Chemical Reagent Co., LTD.

### 2.2 The *In Situ* Immobilization Method of Biohydrogen Slurry

Two immobilization methods, the conventional spherification method and inverse spherification method, were conducted in this work to compare its immobilization effect and releasing property (Bennacef et al., 2021). In the conventional spherification method, SA and biohydrogen slurry are directly mixed and dropped into an aqueous solution containing Ca^2+^. Ca^2+^ penetrates from the outside to the inside, and the outer layer of the “capsule” has a high crosslinking density. In the inverse spherification method, an aqueous solution containing Ca^2+^ is dropped into the mixed solution of sodium alginate and biohydrogen slurry. Ca^2+^ penetrates from the inside to the outside, and the inner layer of the “capsule” has a high crosslinking density (Tsai et al., 2017).

**FIGURE 1:**
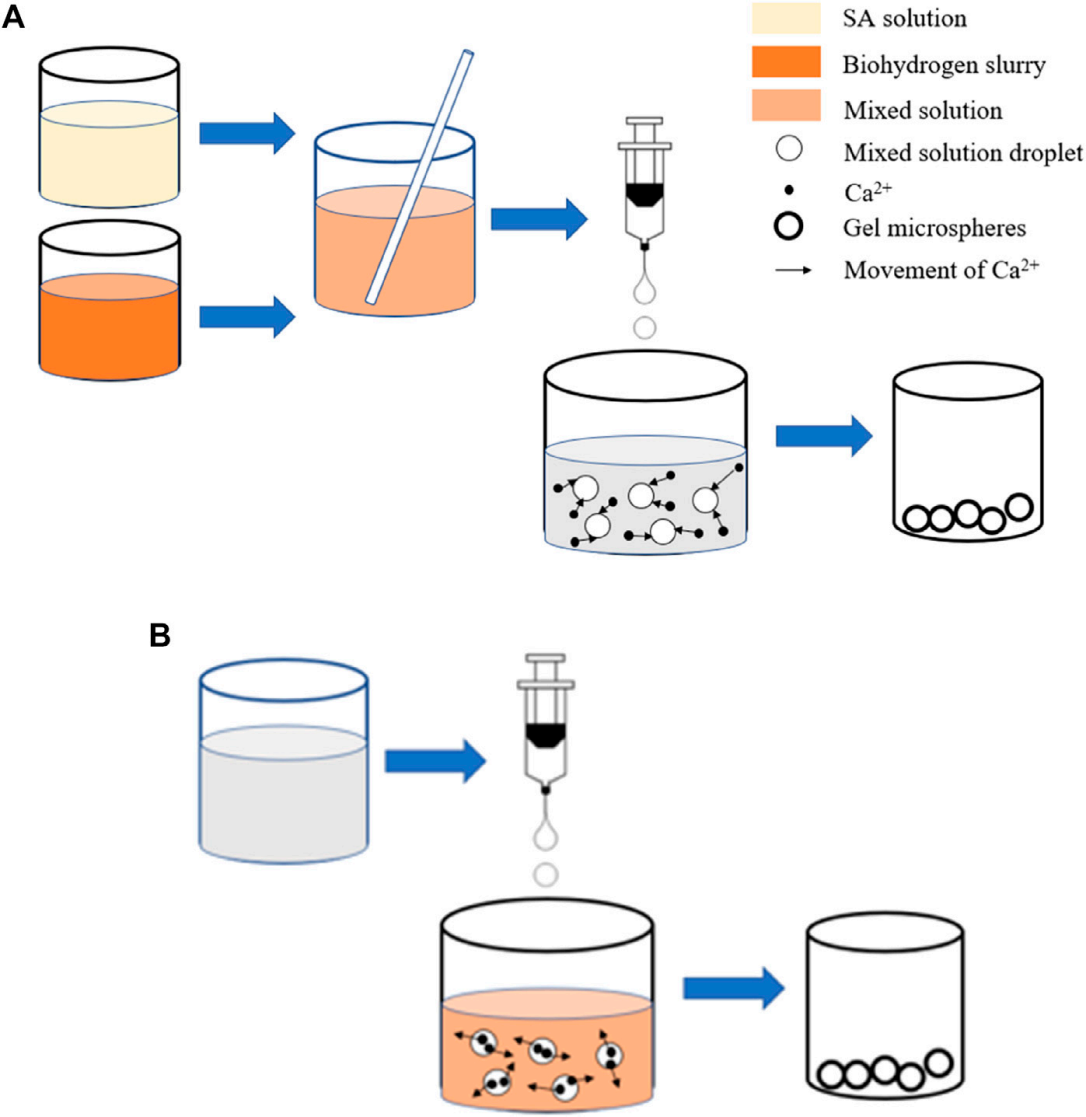
*In situ* immobilization method of SA. **(A)** Conventional spherification. **(B)** Inverse spherification.

Three mL biohydrogen slurry and 7 ml 3.0 wt% SA solution were injected into a 50-ml beaker with a pipette, shaken and oscillated so that the solution was fully mixed. Then, the 10 ml uniform solution was extracted by a 10-ml hypodermic needle and uniformly dropped into a sufficient amount of 10 wt% CaCl_2_ solution. After 2 h, the GMs were filtered out and placed in reserve (Figure 1A); this differs from the conventional spherification method. During the inverse spherification, 10 wt% CaCl_2_ solution was injected into the mixed solution of 3 ml biohydrogen slurry and 7 ml 3.0 wt% SA, and then the next steps were the same as those in the conventional spherification method (Figure 1B).

### 2.3 Immersion Experiment Design

The element release effect and immobilization effect of GM were evaluated by the single-factor experiment. Using the effective volume, a 150-ml cone bottle was used as the release experimental reactor, 12 g GMs were accurately placed in the reactor, and 100 ml deionized water was added to the reactor, sealed with the fresh-keeping film, and the water sample was extracted after static 24 h. In the single-factor experiments, the conditions were changed one by one according to the following levels. Different syringe needle sizes (0.3, 0.7, 1.6, and 3.0 mm), SA concentration (1.0, 1.5, 2.0, 2.5, 3.0, 3.5, and 4.0 wt%), and CaCl_2_ concentration (2, 4, 6, 8, 10, and 12 wt%) were set separately to observe the effect.

### 2.4 Analytical Methods

#### 2.4.1 Immobilization Effect

The molding effect of GM was observed and the number, mass, particle size, and sphericity factor (SF) were also observed. The diameter of the GM was measured with a spiral micrometer. The mass of GM was measured by an electronic balance. The sphericity was calculated according to the following equation (Eq 1):

$$\mathrm{SF}=\frac{d_{max}-d_{min}}{d_{max}+d_{min}},$$ (1)

where *d*_max_ (mm) refers to the maximum diameter of the GM and *d*_min_ (mm) refers to the minimum diameter of the same GM (Benavides et al., 2016). When SF ≤ 0.05, it can be considered to be a standard sphere.

#### 2.4.2 Nutrient Detection

The content of each element is determined by ICP-AES/MS, adding 5 ml nitric acid and 1 ml hydrochloric acid to the bottom of the polytetrafluoroethylene digestion tank for microwave digestion and then the ratio of acid and digestion time was adjusted on the basis of different sample conditions. After digestion, the volume can be fixed to 50 ml. The potassium content was measured by using a flame photometer. The total nitrogen content of the biohydrogen slurry and water sample was determined by a TOC organic carbon total nitrogen analyzer (Jena, N3-1082/AQ, Germany), using oxygen as the carrier gas, control detector temperature was 800 °C, and the sample was 200.0uL per injection. Amino acids were determined by HPLC (Agilent, 1260, United States).

#### 2.4.3 GM Yield (*Y%*) Refers to the Ratio Between GM Output and Liquid Input; *Y%* Is Expressed by the Following Expression (Eq 2) (Chan, 2011)

$$Y\%=\frac{M_{out}}{M_{in}}\times 100,$$ (2)

where *M*_*out*_ is the weight of the GM obtained and *M*_*in*_ is the weight of liquid used.

#### 2.4.4 Encapsulation Efficiency (*EE%*) Refers to the Ratio of the Weight of Elements in GM to the Total Weight of Elements Invested (Eq 3) (Yari et al., 2020):

$$\mathrm{EE}\%=\frac{M_{a}-M_{b}}{M_{a}}\times 100,$$ (3)

where *M*_*a*_ is the weight of the elements added and *M*_*b*_ is the weight of elements lost during immobilization.

#### 2.4.5 Loading Capacity (*LC%)* Refers to the Ratio of the Weight of Elements in GM to the Total Weight of GM (Eq 4) (Gong et al., 2011)

$$\mathrm{LC}\%=\frac{M_{a}-M_{b}}{M_{C}}\times 100,$$ (4)

where *M*_*c*_ is the weight of GM.

### 2.5 Statistical Analysis

All data collation and mapping were performed by using OriginPro 2018C and Excel software. Three groups of parallel experiments were set for all samples, and the immobilization effect of each factor on the biohydrogen slurry and nutrient release effect of GM was analyzed by single-factor standard deviation (Markowski, 1990).

## 3 Results and Discussion

### 3.1 Comparison of the Nutrient Release Effect Between Conventional and Inverse Spherification

**FIGURE 2:**
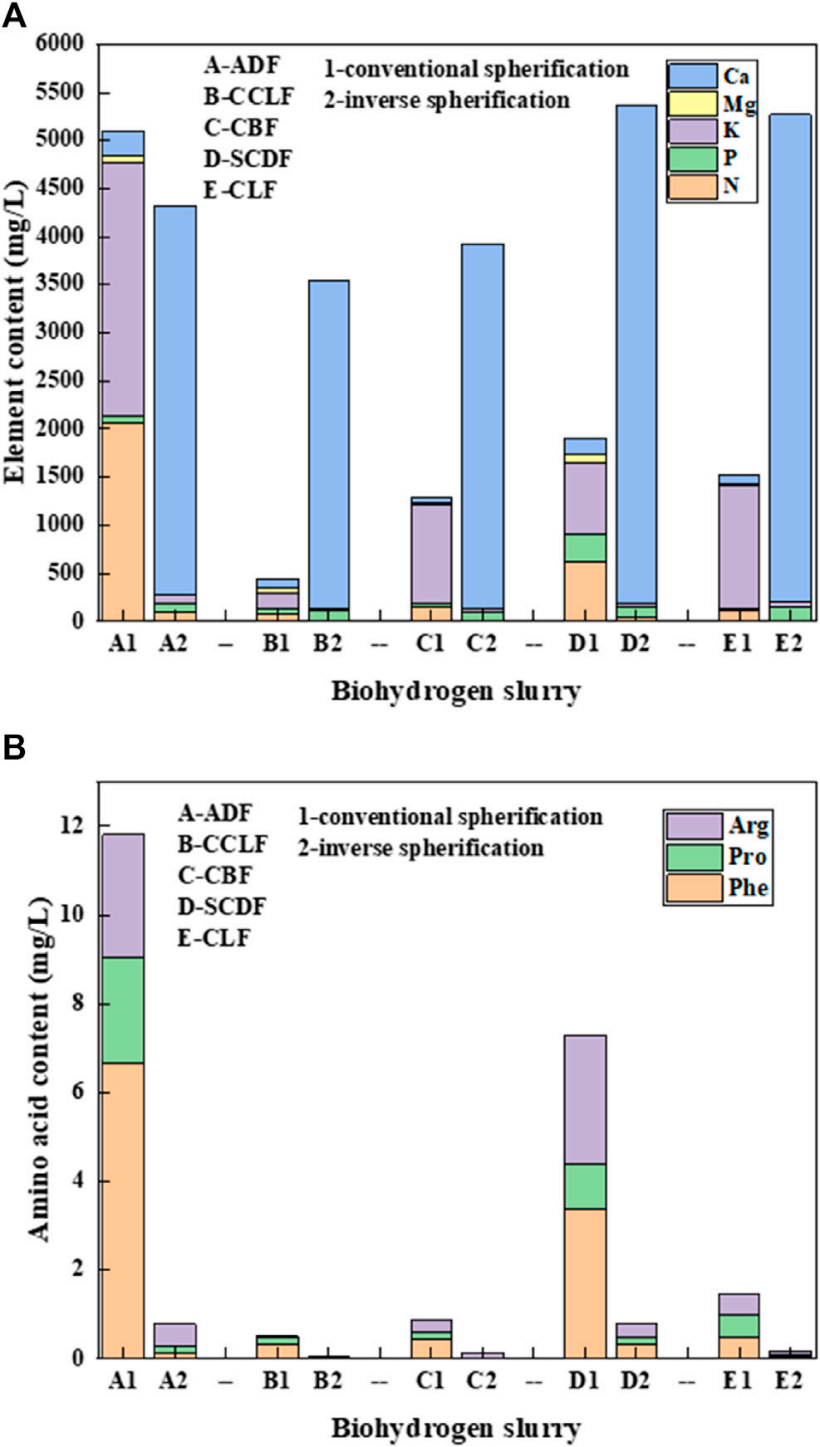
Release effect of nutrients in five kinds of biohydrogen slurry. **(A)** Release effect of nutrient elements. **(B)** Release effect of amino acids.

As shown in Figure 2A, the nitrogen content in the sustained release nutrient solution prepared by conventional spherification is 10–20 times higher than that of inverse spherification, while the phosphorus content is basically similar. The potassium content is 20–30 times higher than that of inverse spherification, and the magnesium content is 5–15 times higher than that of inverse spherification. When using the inverse spherification method, nutrients need to enter the inside of the droplet and then be fixed, while the sodium alginate gel process is very fast, resulting in a large number of nutrients lost in the external solution, making it less nutrient-embedded. The content of calcium in the nutrient solution prepared by inverse spherification is about 60 times higher than that by conventional spherification, but only a fraction of the calcium is released from the anerobic biohydrogen slurry, and most are released by decomposition of the GM’s shell. The faster the calcium release, more volatile the gel forming. It can prove that the GMs prepared by conventional spherification have better a release effect than the glue block formed by inverse spherification.

The contents of main amino acids in the five biohydrogen slurries after slow release are shown in Figure 2B. The content of Phe in conventional spherification was 10–60 times higher than that in inverse spherification. The release of Phe in conventional spherification of the alfalfa biohydrogen slurry even reached 6.661 mg/L, and the content of Pro in conventional spherification was more than 10 times higher than that in inverse spherification. The content of Arg was 2–10 times higher in conventional spherification than in the inverse method. It can be clearly seen that the GMs produced by the conventional spherification had much higher amino acid content in the nutrient solution that is released than the glue block produced by the inverse spherification.

The utilization rate of nutrient elements and amino acids in biohydrogen slurry was significantly improved by conventional spherification (*p* = 0.02542 < 0.05), and the high value immobilized treatment means would provide some positive significance for the clean utilization of biohydrogen slurry. The conventional spherification method was used for the subsequent optimization experiments.

### 3.2 Effects of GM Sizes on the Immobilization and Release of Nutrients

Generally, the immobilization effect of GM was evaluated by sphericity, EE%, LC%, and Y%, and the release effect of GM was evaluated by the release rate of nutrients. The size of the GM was adjusted by changing the syringe needle size, as shown in Table 2. Four syringe needles correspond to four particle sizes. It can be clearly seen that only when the syringe needle size was 1.6 mm, the SF of GM is <0.05, which was more similar to spheres than other kinds. This may be because the liquid dropped faster when the syringe needle was small, and the impact force was too large when it came in contact with the CaCl_2_ solution, resulting in irregular GM formation; when the syringe needle was too large, the droplet volume and gravity became larger and the antideformation ability was weak, which increased SF. GM size had little influence on the EE% and LC% of N and K. When the needle was 1.6 mm, Y% was the highest. When GM size was small, the specific surface area increased, then the proportion of the gel shell increased, and the water loss was too much, leading to the decrease of GM total mass; when GM size was large, the amount of GM produced by a certain amount of biohydrogen slurry decreased and the total mass decreased.

**TABLE 2:** Effects of GM with different sizes on the immobilization of nutrients.

Syringe needle size (mm)	GM size (mm)<	SF	EE% (N)	LC% (N)	EE% (K)	LC% (K)	Y%
0.3	2.096	0.078	34.65	0.0545	29.29	0.0507	60.87
	±0.044		±0.15		±0.07		
0.7	2.482	0.062	34.32	0.0491	30.30	0.0477	66.96
	±0.089		±0.18		±0.06		
1.6	3.182	0.045	34.56	0.0472	30.30	0.0455	70.16
	±0.010		±0.06		±0.11		
3.0	3.505	0.057	34.51	0.0502	27.27	0.0419	68.59
	±0.075		±0.07		±0.03		

**FIGURE 3:**
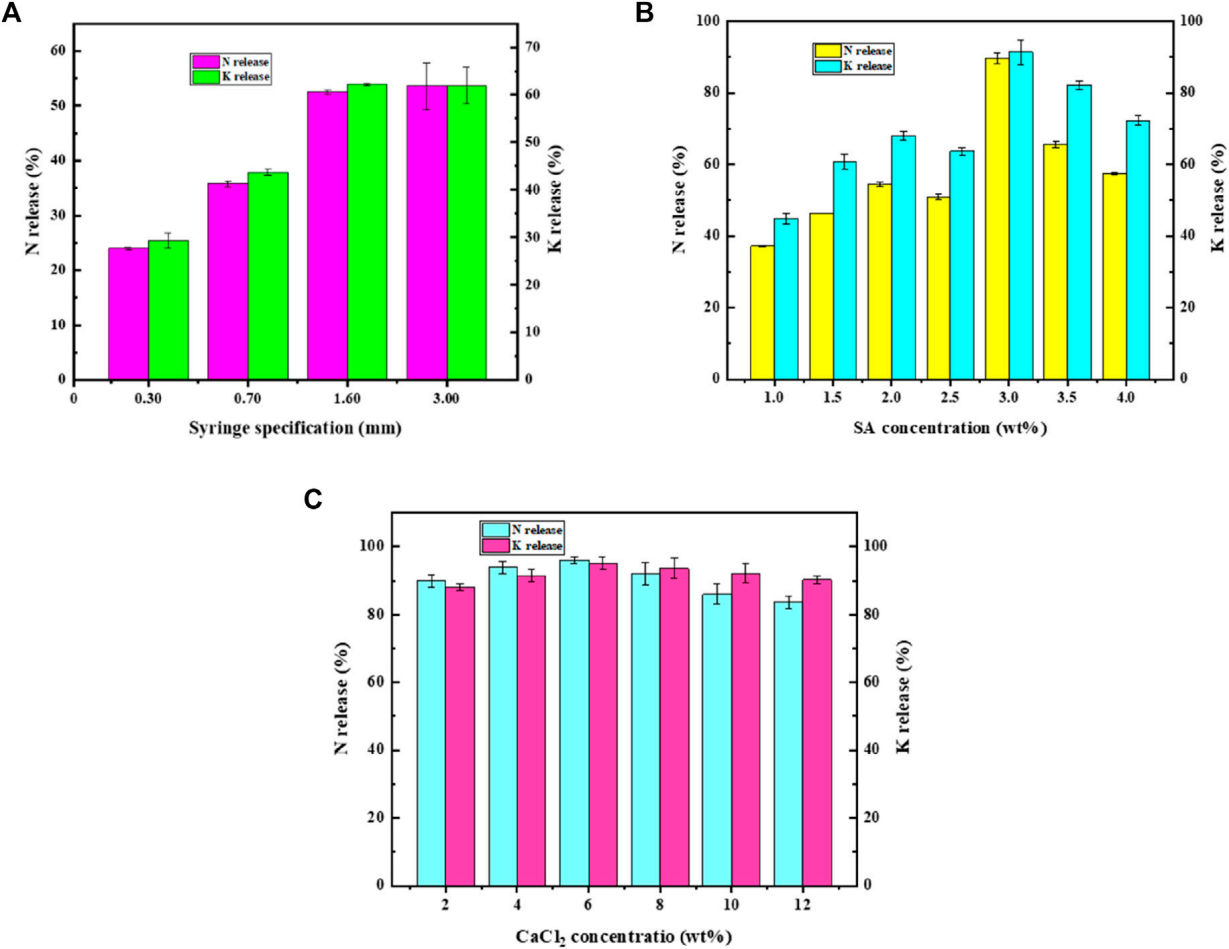
Effects of different factors on the release of nutrients. **(A)** Sizes of GM. **(B)** SA concentration. **(C)** CaCl_2_ concentration.

As shown in Figure 3A, the cumulative release of N and K for GM prepared with 0.3 and 0.7 mm needles was much lower than that prepared with 1.6 and 3.0 mm needles. The cumulative release of N and K of GM prepared with 1.6 and 3.0 mm needles was about 53 and 62%, respectively, showing no significant difference (P_N_ = 0.06759 > 0.05 and P_K_ = 0.07090 > 0.05). Therefore, it is a good choice to use a 1.6-mm syringe needle to prepare GM.

### 3.3 Effect of SA Concentration on Immobilization and Nutrient Release of GM

As the material is directly carrying biohydrogen slurry, SA concentration plays an important role in GM molding. As shown in Table 3, with the increase of SA concentration, the sphericity of GM gradually decreased. When the SA concentration reached 3.0 wt% SA, GM had approximated to a sphere. When SA concentration was high in the droplet, the egg-box structure formed by crosslinking with Ca^2+^ was more stable and had stronger impact resistance. The EE% and LC% of GM prepared by 1% and 1.5% SA were slightly higher than those of other concentrations, but there was no significant difference (P_EE%_ = 0.14458 > 0.05 and P_LC%_ = 0.23465 > 0.05). The Y% of 3, 3.5, and 4.0 wt% SA was obviously higher than that of low concentration SA.

**TABLE 3:** Effects of SA concentration on the immobilization of nutrients.

SA wt%	SF	EE% (N)	LC% (N)	EE% (K)	LC% (K)	Y%
1.0	0.141	37.84	0.0653	31.31	0.0595	55.44
		±0.11		±0.15		
1.5	0.073	36.91	0.0502	30.81	0.0461	70.39
		±0.17		±0.27		
2.0	0.055	35.39	0.0476	29.29	0.0433	71.21
		±0.03		±0.31		
2.5	0.053	36.38	0.0496	30.81	0.0462	70.28
		±0.04		±0.07		
3.0	0.049	34.96	0.0420	28.79	0.0380	79.74
		±0.14		±0.12		
3.5	0.018	35.99	0.0434	27.27	0.0362	79.38
		±0.07		±0.00		
4.0	0.017	36.83	0.0463	27.78	0.0384	76.19
		±0.10		±0.27		

The cumulative release of N and K increased gradually in the low concentration of SA and reached a peak at 3.0 wt%, and then the cumulative release gradually decreased (Figure 3B). Combined with the experimental process, the result was caused by the slow gel forming process of SA with low concentration and excessive loss of nutrients. When the SA concentration is too high, the egg-box structure proportion in GM is too large and the porosity decreases, resulting in insufficient release of nutrients. Hence, the SA concentration of 3.0 wt% is more suitable for embedding the biohydrogen slurry.

### 3.4 Effect of CaCl_2_ Concentration on Immobilization and Nutrient Release of GM

CaCl_2_ concentration is also an important factor affecting *in situ* immobilization of the biohydrogen slurry (Table 4). With the increase of CaCl_2_ concentration, the SF of GM increased, but when the concentration is 8 wt%, SF had exceeded 0.05. This was because with the increase of CaCl_2_ concentration, the surface tension of the aqueous solution would also increase, and the resistance of the droplet when falling to the water surface would increase, resulting in serious deformation of GM. EE% of N and K also increased with increasing CaCl_2_ concentration, but the range of change was not large. This may be because when the concentration of Ca^2+^ in the solution was high, the external osmotic pressure increased, and the loss of nutrients in GM would be reduced. The variation of LC% and Y% was relatively small. These phenomena indicate that the increase of CaCl_2_ concentration had a slight positive effect on the immobilization effect of GM.

**TABLE 4:** Effects of CaCl_2_ concentration on the immobilization of nutrients.

CaCl_2_ wt%	SF	EE% (N)	LC% (N)	EE% (K)	LC% (K)	Y%
2	0.038	35.44	0.0493	30.55	0.0479	76.61
		±0.01		±0.02		
4	0.044	36.09	0.0463	30.81	0.0434	74.71
		±0.94		±0.25		
6	0.047	38.37	0.0476	32.32	0.0438	77.68
		±0.59		±0.15		
8	0.061	38.43	0.0482	32.82	0.0439	76.28
		±0.27		±0.12		
10	0.062	40.81	0.0486	33.33	0.0437	80.33
		±0.16		±0.01		
12	0.068	42.42	0.0512	33.84	0.0449	79.29
		±0.90		±0.31		

As can be seen from Figure 3C, with the change of CaCl_2_ concentration, the cumulative release of N was maintained between 83.62 and 96.02%, and the cumulative release of K was maintained between 88.13 and 95.08%, with the change range within 15%. There was an inflection point at 6 wt%, and the cumulative release was the largest at this time. Considering the immobilization effect and release effect, 6 wt% CaCl_2_ is suitable as the embedding carrier.

## 4 Conclusion

Previous research on the treatment of biological mud does not have a very good effect; this study will use biological mud with higher efficiency. In this work, the SA immobilization method was proposed to prepare the “slurry capsule” by biohydrogen slurry produced from FHP. Good pellet immobilization ability and the release level of nutrient elements are shown. It not only realized the harmless treatment of biohydrogen slurry and protected the environment but also achieved the purpose of resource utilization of biohydrogen slurry and solved an issue concerning the clean production of hydrogen energy.

The immobilization effect of conventional spherification is obviously better than that of the inverse spherification. The mixture (3.0 wt% SA/biohydrogen slurry) dropped into 6 wt% CaCl_2_ solution by a 1.6-mm syringe needle, which was found to be the optimal immobilization condition. The sphericity factor, nitrogen release, yield, nitrogen encapsulation efficiency, and nitrogen loading capacity reached to 0.047, 96.20, 77.68, 38.37, and 0.0476%, respectively.

Compared with previous studies, this is a cleaner immobilization method of biohydrogen slurry, which can ensure the utilization value of fermentation tail liquid and provide some favorable directions for environmental protection and waste resource utilization research. This study provides new ideas for ecological conservation and carbon neutrality, which is of great significance for sustainable development.

## Data Availability

The raw data supporting the conclusion of this article will be made available by the authors, without undue reservation.
